# Secretion systems shaping host–pathogen interactions in *Burkholderia* species

**DOI:** 10.1007/s00253-026-13833-x

**Published:** 2026-05-22

**Authors:** Sparsha Pallen, Aishwariya Madhavan, Sivakumar Subhashree, Varshith M.R., Piyush Behari Lal, Chiranjay Mukhopadhyay, Roberto Herai, Somasish Ghosh Dastidar, Ranita Ghosh Dastidar

**Affiliations:** 1 Present Address: Department of Biochemistry, Kasturba Medical College, Manipal Academy of Higher Education, Manipal, Karnataka 576104 India; 2 Department of Microbiology, Kasturba Medical College, Manipal Academy of Higher Education, Manipal, Karnataka 576104 India; 3 Department of Anatomy, Kasturba Medical College, Manipal Academy of Higher Education, Manipal, Karnataka 576104 India; 4 Graduate Program in Health Sciences, School of Medicine and Life Sciences, Pontifícia Universidade Católica do Paraná, Paraná, India

**Keywords:** *Burkholderia* species, Host response, Pathogenesis, Type secretion system, T3SS, T6SS

## Abstract

**Abstract:**

Understanding the virulence mechanisms of pathogenic bacteria is crucial for elucidating their ability to cause disease. Among these, type secretion systems (TSS) are important for mediating host-pathogen interactions. This review focuses on the TSS in *Burkholderia* species, emphasizing their molecular structures, mechanisms, and their role in virulence. *Burkholderia* spp. employ a variety of TSS, each contributing distinctly to pathogenicity. Type 1 SS (T1SS) facilitate the export of toxins/enzymes to the extracellular space, promoting host-tissue damage and immune modulation. T2SS secretes hydrolytic enzymes that breach the host barriers. T3SS injects the effectors directly into the host cell, thereby aiding intracellular bacterial survival. T4SS translocates proteins and DNA, disrupting the host immune defenses and delaying vacuolar maturation. T5SS autotransporters mediate adhesion, invasion, and intracellular mobility through actin polymerization. T6SS targets both host and competing microbes through delivering toxic effectors, aiding in immune evasion, competition, and replication. Comparatively, T7SS–T11SS are discussed to provide a broader bacterial context as these TSSs are not established in *Burkholderia* spp. currently. This review uniquely integrates the structural, functional, and synergistic roles of all major secretion systems (Types 1–6) in *Burkholderia* spp., highlighting how their coordinated actions collectively shape host-pathogen interactions and drive virulence.

**Key points:**

• *Burkholderia spp. exploit diverse secretion systems to manipulate host cells to enable their intracellular survival and immune evasion.*

• *This review uniquely integrates secretion systems with host actin remodeling, outer membrane vesicles, biofilm formation, and antimicrobial resistance to present a systems-level view of Burkholderia-associated infection pathogenesis.*

• *We highlight emerging diagnostic, therapeutic, and vaccine-relevant targets to address Burkholderia spp. infections and advance global health goals (Sustainable Development Goal 3).*

## Introduction

The *Burkholderia* genus is composed of a diverse group of Gram-negative, majorly oxidase-positive, non-sporulating aerobic bacilli within the Betaproteobacteria class, known for its environmental survival and adaptation to various hosts (Stopnisek et al. 2014). Initially classified under *Pseudomonas* DNA homology group II, these organisms were later reassigned to an independent genus based on distinct genetic and phenotypic characteristics (Hotta et al. 1992; Eberl and Vandamme 2016). Clinically relevant *Burkholderia* spp. are broadly grouped into *Burkholderia pseudomallei*, the *Burkholderia cepacia* complex (Bcc), and other pathogenic *Burkholderia* spp. (West et al. 2008; Inglis and Merritt 2014).

**Fig. 1:**
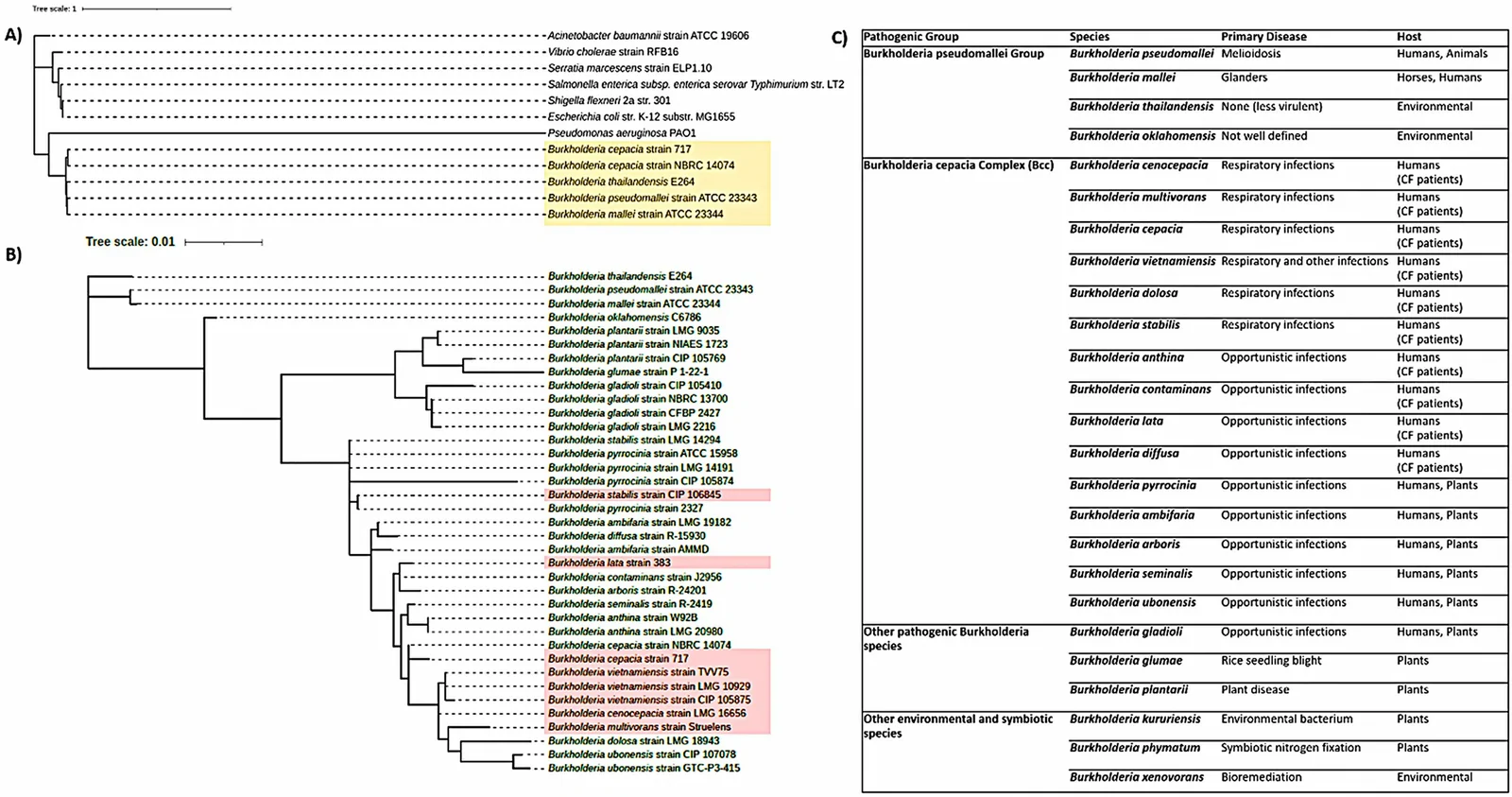
Phylogenetic tree. **A** Phylogenetic tree representation comparing *Burkholderia* species with their closest relation, *Pseudomonas aeruginosa*, and other well-known Gram-negative bacteria. **B** Phylogenetic tree representation comparing all groups of *Burkholderia* species. **C** Tabular representation of all groups of *Burkholderia* species with their primary disease and host. Table 1 elaborates specific clinical characteristics of all these groups

Several *Burkholderia* spp. are highly virulent human pathogens (Fig. 1 and Table 1). *B. pseudomallei* causes melioidosis, a potentially fatal infection endemic to tropical regions and can cause acute or chronic pneumonia, septic shock, abscess formation, and bacteremia, with high mortality if untreated (White 2003; Meumann et al. 2023). *Burkholderia mallei* causes glanders and primarily infects equids but can also cause severe zoonotic disease in humans, including ulcerative lesions, lymphadenopathy, and respiratory involvement (Singha et al. 2021; Waag et al. 2021; Dehghan Rahimabadi et al. 2023). The Bcc comprises over 20 closely related species, notably *B. cenocepacia*, *B. multivorans*, *B. vietnamiensis*, *B. cepacia*, and *B. dolosa*, which are major causes of Cepacia syndrome in cystic fibrosis (CF) patients, leading to progressive lung disease and increased mortality (Mahenthiralingam et al. 2008; Daccò et al. 2023). Although not a classical CF pathogen, *Burkholderia pseudomallei* in rare instances has also been isolated from CF patients, particularly those with a history of travel or residence in endemic regions. Chronic infection poses serious therapeutic challenges due to persistent inflammation and antibiotic resistance (Corral et al. 2008).

**Table 1:** Pathogenic groups of *Burkholderia* species with emphasis on primary disease, host and characteristics

Pathogenic group	Species	Primary disease	Host	Characteristics	Reference
*Burkholderia pseudomallei* group	***Burkholderia pseudomallei***	Melioidosis	Humans, animals	Found in soil and water in tropical and subtropical regions, causes severe and potentially fatal infections. Known for its potential as a bioterrorism agent due to high virulence	(Birnie et al. 2022; Currie 2026)
	***Burkholderia mallei***	Glanders	Horses, humans	Primarily affects horses but can infect humans. Known for its potential as a bioterrorism agent due to high virulence	(Sukmanadi et al. 2025)
	***Burkholderia thailandensis***	None (less virulent)	Environmental	Found in the same niches as *B. pseudomallei*. Used as a model organism for studying melioidosis	(Haraga et al. 2008; Hantrakun et al. 2018)
	***Burkholderia oklahomensis***	Not well defined	Environmental	Isolated from the environment and human clinical samples in the United States. Pathogenic potential in humans not well understood	(Glass et al. 2006; Dashti et al. 2026)
*Burkholderia cepacia* complex (Bcc)	***Burkholderia cenocepacia***	Respiratory infections	Humans (CF patients)	Major pathogen in cystic fibrosis (CF) patients. Known for causing rapid lung function decline and increased mortality	(Gutiérrez Santana and Coria Jiménez 2024)
	***Burkholderia multivorans***	Respiratory infections	Humans (CF patients)	Commonly isolated from CF patients. Causes chronic lung infections and can lead to significant morbidity	(Lood et al. 2021; Daccò et al. 2023)
	***Burkholderia cepacia***	Respiratory infections	Humans (CF patients)	The type species of the complex. Notorious for causing “*cepacia* syndrome,” a rapid and often fatal decline in lung function in CF patients	(Daccò et al. 2023; Gutiérrez Santana and Coria Jiménez 2024)
	***Burkholderia vietnamiensis***	Respiratory and other infections	Humans (CF patients)	Less common but still significant in CF patients. Can cause various infections, including lung and bloodstream infections	(Cescutti et al. 2013; Kar et al. 2023; Flores-Vega et al. 2023; Daccò et al. 2023)
	***Burkholderia dolosa***	Respiratory infections	Humans (CF patients)	Causes persistent lung infections in CF patients. Associated with high rates of morbidity and mortality	(Biddick et al. 2003; Moreira et al. 2014; Roux et al. 2017)
	***Burkholderia stabilis***	Respiratory infections	Humans (CF patients)	Part of the *B. cepacia* complex with similar pathogenic traits. Involved in chronic infections in CF patients	(Seth-Smith et al. 2019)
	***Burkholderia anthina***	Opportunistic infections	Humans (CF patients)	Found in CF patients, causing chronic respiratory infections Exhibits antibiotic resistance, complicating treatment	(León et al. 2023)
	***Burkholderia contaminans***	Opportunistic infections	Humans (CF patients)	Emerging pathogen in CF patients. Known for its resistance to multiple antibiotics	(León et al. 2023)
	***Burkholderia lata***	Opportunistic infections	Humans (CF patients)	Causes respiratory infections in CF patients. Shares many characteristics with other Bcc species	(Leong et al. 2018)
	***Burkholderia diffusa***	Opportunistic infections	Humans (CF patients)	Less commonly isolated but still pathogenic in CF patients Exhibits resistance to several antibiotics	(Vanlaera et al. 2008)
	***Burkholderia pyrrocinia***	Opportunistic infections	Humans, plants	Isolated from CF patients and environmental sources Can cause infections in immunocompromised individuals	(Savi et al. 2014)
	***Burkholderia ambifaria***	Opportunistic infections	Humans, plants	Known for plant growth-promoting properties. Can cause opportunistic infections in immunocompromised patients	(Goodlet et al. 2019)
	***Burkholderia arboris***	Opportunistic infections	Humans, plants	Found in both human clinical samples and environmental sources. Capable of causing infections in CF patients	(Vanlaera et al. 2008)
	***Burkholderia seminalis***	Opportunistic infections	Humans, plants	Isolated from both human infections and plant environments. Can cause opportunistic infections in immunocompromised individuals	(Vanlaera et al. 2008)
	***Burkholderia ubonensis***	Opportunistic infections	Humans, plants	Isolated from CF patients and environmental sources. Capable of causing infections in immunocompromised individuals	(Somprasong et al. 2021a)
Other pathogenic *Burkholderia* species	***Burkholderia gladioli***	Opportunistic infections	Humans, plants	Causes respiratory infections in CF patients and is a plant pathogen. Known for its resistance to multiple antibiotics	(Quon et al. 2011)
	***Burkholderia glumae***	Rice seedling blight	Plants	Plant pathogen causing rice seedling blight. Can be an opportunistic pathogen in immunocompromised humans	(Singh et al. 2024)
	***Burkholderia plantarii***	Plant disease	Plants	Causes seedling disease in plants. Not typically associated with human infections	(Mannaa et al. 2024)

Pathogenic *Burkholderia* spp. are highly resilient and intrinsically resistant to antimicrobial pressure (Fig. 1C). This resilience is driven in part by multidrug efflux pumps of the resistance-nodulation-division (RND) family, including the well-characterized systems AmrAB-OprA, BpeAB-OprB, and BpeEF-OprC in *Burkholderia pseudomallei*, which are key determinants of intrinsic and acquired multidrug resistance (Somprasong et al. 2021b; Meumann et al. 2023). Structural studies further support the role of these transporters (e.g., BpeB and BpeF) in active antibiotic efflux (Kato et al. 2023). In the *Burkholderia cepacia* complex (Bcc), multiple genomically defined RND efflux systems, including RND-3, RND-4, and RND-10, have been identified and are conserved across species, contributing to comparable multidrug resistance phenotypes (Somprasong et al. 2021b). In addition to efflux pumps, these organisms also possess a relatively impermeable outer membrane, antibiotic-degrading enzymes, and the ability to adopt metabolically dormant persister states (Nierman et al. 2004; Nierman et al. 2015; Somprasong et al. 2021b). Furthermore, these organisms form biofilms, employ quorum sensing, produce capsular polysaccharides, proteases, and lipases, and deploy specialized protein secretion systems to deliver virulence factors directly into host cells (Bzdyl et al. 2022).

Because of their high virulence, aerosol infectivity, and potential for misuse, *B. pseudomallei* and *B. mallei* are regulated as Tier 1 Select Agents by the U.S. Federal Select Agent Program and are classified as Risk Group 3 pathogens requiring high-containment handling in India and elsewhere (Grund et al. 2021; Varshith et al. 2024).

Protein secretion is essential for Gram-negative bacterial pathogenesis. Because Gram-negative bacteria have a double-membrane cell envelope with a thin peptidoglycan layer and an outer membrane containing lipopolysaccharides, they require specialized secretion systems to transport proteins across this complex barrier (Yeh et al. 2024). Consequently, Gram-negative bacteria have evolved multiple secretion systems, including T1SS-T6SS (Type I–VI Secretion Systems) and T8SS–T11SS (Type VIII–XI Secretion Systems) (Table 2). It is important to note that T8SS and T9SS have not been reported or experimentally established in *Burkholderia* spp., and their inclusion here reflects general bacterial classification rather than organism-specific presence. T7SS is predominantly described in Gram-positive bacteria, but homologous systems have recently only been predicted in some Gram-negative lineages, including Proteobacteria (Tran et al. 2021; Famelis et al. 2023).

**Table 2:** Overview of the salient features of bacterial secretory systems

Secretory system	Features	References
T1SS	T1SS is a one-step protein secretion system in Gram-negative bacteria that transports toxins and other virulence factors directly from the cytoplasm to the extracellular space. T1SS commonly secretes virulence factors such as HlyA in *Escherichia coli*, HasA/HasAp hemophores in *Serratia* and *Pseudomonas*, and hemolysins in *Burkholderia*, all of which contribute to host cell lysis, immune evasion, and iron acquisition	(Hodges et al. 2023)
T2SS	T2SS is a dual step secretion system in Gram-negative bacteria which delivers folded proteins from the periplasm to the outer membrane, typically after they are first transported via the Sec or Tat pathways. They commonly secretes hydrolytic enzymes like proteases, phospholipases, collagenases, lipases and virulence factors like immunogenic proteins such as MprA and BLF1, associated in tissue degradation and immune modulation in the host cells	(Naskar et al. 2021)
T3SS	T3SS is a single step protein translocation apparatus that functions as a molecular syringe, injecting effector proteins directly into host cells, bypassing the periplasm, manipulating cellular processes and promoting intracellular survival in the host. Factors secreted include effectors, translocators, and regulatory proteins and they are also generally considered a hallmark of pathogenicity thus serve as promising therapeutic target for therapeutics due to its essential role in bacterial cell invasion, immune modulation, and long-term survival	(Wallner 2021)
T4SS	T4SS are not exclusive to Gram-negative bacteria; they are also present in Gram-positive bacteria. They primarily operate as a uni-step mechanism, involves the transfer of substrates such as proteins or DNA from the bacterial cytoplasm to the outside of the cell or into recipient cells directly without a periplasmic intermediate. However, some T4SSs also function via a two-step process, where substrates first enter the periplasm before being secreted. They also play a significant role in horizontal gene transfer and pathogenicity	(Zhang et al. 2009; Macé and Waksman 2024)
T5SS	T5SS, also known as the autotransporter system, is *utilized* by Gram-negative bacteria to transport proteins across their outer membrane. These proteins, called autotransporters, typically consist of a β-barrel domain that embeds in the outer membrane and a passenger domain that is transported outside the cell to perform functions such as adhesion, invasion, or enzymatic activity	(Stevens et al. 2005; Nicolay et al. 2015)
T6SS	T6SS is a complex, contractile nanomachine found in many Gram-negative bacteria and operates via a one-step mechanism, allowing the direct delivery of toxic effectors into the surrounding prokaryotic or eukaryotic cells. These effectors include enzymes like phospholipases, proteases, and nucleases that commonly compromise the host cell integrity and support both interbacterial competition and pathogenesis	(Nguyen et al. 2018; Cherrak et al. 2018; Lennings et al. 2019)
T7SS	T7SS is a specialized protein export mechanism found in Gram-positive bacteria, notably in genera such as *Staphylococcus* and *Mycobacterium*. It facilitates the secretion of effector proteins across their complex cell envelopes, involved in bacterial virulence and interbacterial interactions	(Spencer and Doran 2022)
T8SS	T8SS, also known as the extracellular nucleation-precipitation pathway, is utilized by certain Gram-negative bacteria to assemble and secrete amyloid fibers called curli. Curli fibers are proteinaceous appendages that play a role in surface adhesion, biofilm formation, and interactions with host cells, described in E*scherichia coli*	(Wittmers et al. 2022)
T9SS	T9SS is restricted so far to certain Gram-negative bacteria of the phylum Bacteroidetes and facilitates the secretion of enzymes that degrade complex polysaccharides	(Gorasia et al. 2020)
T10SS	T10SS is distinct from other secretion systems due to holin-mediated lysis for protein export, identified in certain Gram-negative bacteria, including *Salmonella enterica, Yersinia entomophaga* and *Serratia marcescens*. Holin/endolysin/spanin clusters outside bacteriophages were recently identified as T10SS, which were proposed to function as an avenue for lytic and non-lytic protein export	(Sitsel et al. 2024)
T11SS	T11SS is present in the Gram-negative phylum Proteobacteria, including many human pathogens such as *Neisseria meningitidis, Acinetobacter baumanii, Haemophilus haemolyticus,* and *Proteus vulgaris*	(Grossman et al. 2024)

*Burkholderia* spp. deploy multiple secretion systems that collectively drive virulence and intracellular survival. These include T1SS and T2SS for secretion of toxins and degradative enzymes (Morgan et al. 2017; DeShazer 2019; Korotkov and Sandkvist 2019; Naskar et al. 2021; Hodges et al. 2023), T3SS for host cell manipulation and intracellular persistence (Choh et al. 2021), and T4SS–T6SS for modulation of phagosome maturation, immune evasion, intracellular replication, and cell-to-cell spread (Juhas et al. 2008; Cherrak et al. 2018; Lennings et al. 2019; Dautin 2021).

This review highlights the diversity and functional significance of all TSS in *Burkholderia* spp. pathogenesis, particularly in host interaction and intracellular survival. Previous reviews have primarily focused on individual secretion systems or virulence traits in isolation, reflecting a key limitation in the field. This has resulted in a critical gap in integrating secretion systems with host–pathogen interactions and antimicrobial resistance mechanisms. The novelty of this review lies in providing a unified framework that links secretion systems with host actin dynamics, outer membrane vesicles (OMVs), biofilm formation, and multidrug resistance, thereby addressing these existing gaps. It also provides a comprehensive overview of *Burkholderia* spp. pathogenesis relevant to global health and Sustainable Development Goal 3. Future studies integrating structural biology, proteomics, and genomics are essential for identifying novel virulence factors, improved diagnostics, and vaccine targets to combat *Burkholderia*-associated diseases and mortality.

### Classification of bacterial secretion systems

All bacterial secretion systems can be broadly classified into classes (Class I–IV) based on their mode of translocation (Christie 2019). Classes I–III are classified by the systems in Gram-negative bacteria, while Class IV is found in Gram-positive bacteria. Class I systems consist of T5SS, the chaperone-usher pathway, and T8SS, which are restricted to the outer membrane and rely on a two-step secretion mechanism via the Sec or Tat pathways which are largely ATP independent, primarily contributing to adhesion, pilus assembly, biofilm formation, and host tissue colonization. Class II systems, consisting of T2SS, type IV pili (T4P) (due to the shared history with T2SS), and T9SS, span across both membranes but export substrates across the outer membrane only. In contrast to Class I, these are dependent on ATP hydrolysis and proton motive force; these systems facilitate enzyme secretion, motility, immune evasion, and pathogenesis (Saak and Gibbs 2016). Class III secretion systems include T1SS, T3SS, T4SS, and T6SS, which enable single-step translocation of substrates across both membranes, often directly into host or competitor cells. Among these, T3SS and T6SS have evolved specialized injection-like structures and are strongly associated with bacterial virulence, host manipulation, and interbacterial competition (Saak and Gibbs 2016). Finally, Class IV systems, exemplified by T7SS in Gram-positive bacteria such as *Mycobacterium* spp., mediate protein secretion across the cytoplasmic membrane and thick cell wall, contributing to virulence, metal ion uptake, biofilm formation, DNA exchange, and passive sliding motility. Recently described secretion systems, including T10SS and T11SS, remain under active investigation. T10SS is predominantly reported in Gram-positive bacteria and shares similarities with T7SS, whereas T11SS in Gram-negative bacteria appears to be functionally related to T9SS-mediated protein transport (Suparak et al. 2005).

### Type 1 secretion system

**Fig. 2:**
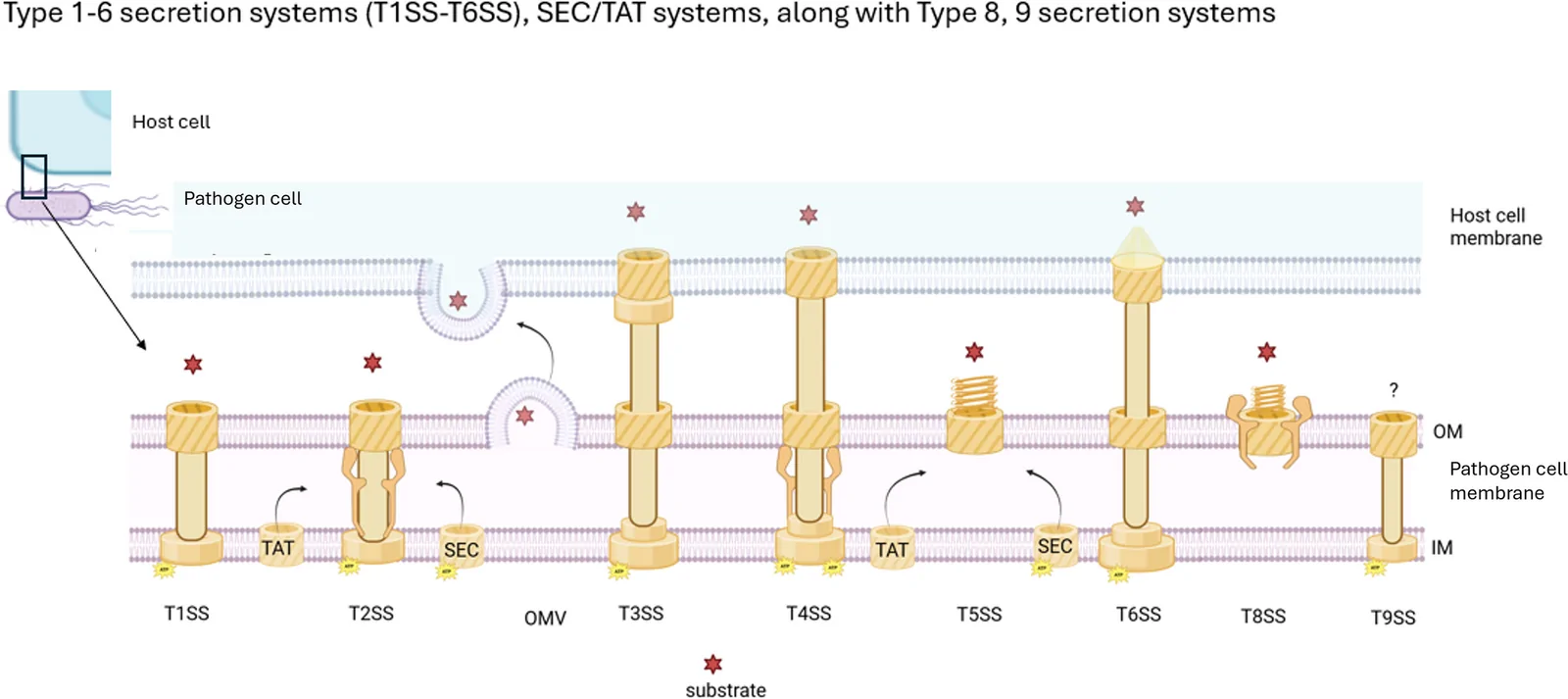
Schematic representation of the Type 1–6 secretion systems (T1SS–T6SS), SEC/TAT systems, along with Type 8, 9 secretion systems (not currently known to be used). Secretion systems are classified based on their mechanism of substrate translocation: single-step systems (T1SS, T3SS, T4SS, T6SS) transport substrates directly across both membranes in one step, while multi-step systems (T2SS, T5SS, T8SS) are assisted by SEC/TAT systems to facilitate transport through the inner membrane into the periplasmic space

The Type I Secretion System (T1SS) is a one-step trans-envelope secretion pathway in Gram-negative bacteria to transport substrates directly from the bacterial cytoplasm to the extracellular environment or host cell interface without a periplasmic intermediate (Fig. 2) (Hodges et al. 2023). The T1SS secretion machinery comprises three key components: (i) inner-membrane ATP-binding cassette (ABC), for secretion signal recognition; (ii) ATP-driven energy supply through a membrane-fusion protein (MFP) that bridges the inner and outer membranes; and (iii) TolC-like outer-membrane protein forming the terminal exit duct (Anlauf et al. 2024) (Figs. 2 and 4). T1SS substrates commonly carry a C-terminal “repeats-in-toxin” (RTX) domain which facilitates their efficient recognition and translocation (Thomas et al. 2014; Morgan et al. 2017). This assembly forms a continuous channel that allows the direct, one-step export of unfolded RTX-family toxins from the cytoplasm to the extracellular space, bypassing the periplasm. These RTX toxins possess C-terminal glycine-aspartate (GG) repeats that bind calcium extracellularly, triggering toxin folding and membrane pore formation, which leads to host cell lysis, immune modulation and virulence (Pourhassan et al. 2022).

In *Burkholderia* spp., Type 1 secretion systems (T1SS) (Table 3) play a key role in virulence and pathogenicity through their classic tripartite architecture: an inner membrane ABC ATPase (e.g., HlyB homolog); a membrane fusion protein (MFP, e.g., HlyD homolog); and an outer membrane beta-barrel porin (e.g., TolC homolog) that exports large substrates such as hemolysins, proteases, or lipases directly from the cytoplasm to the extracellular space without periplasmic intermediates (Harland et al. 2007; Yu et al. 2025). In *B. pseudomallei*, major T1SS contributors to nutrient uptake and virulence include multicomponent ABC transporters that export hemolysin-like toxins to lyse host cells, damage tissues, and modulate host-microbial responses (Harland et al. 2007). These systems also facilitate uptake of polyamines, amino acids, and monosaccharides under nutrient stress, with the BPSS0140-BPSS0142 cluster confirmed as a fructose-specific importer via structural modeling and growth assays in mutants (How et al. 2022). In addition, under nutrient limitations, particularly iron restriction, *Burkholderia* spp. rely on high-affinity iron acquisition systems to sustain growth and infection. Siderophores such as ornibactin and pyochelin are secreted to chelate iron from host sources, and the ability to acquire iron is essential for bacterial survival and the establishment of disease, thereby directly linking nutrient acquisition to virulence (Tuanyok et al. 2005; Tuanyok et al. 2008; Harland et al. 2007; Hatcher et al. 2015; Butt and Thomas 2017). In this context, T1SS-associated ABC transport systems contribute to the overall transport capacity of the bacterium, supporting metabolic adaptation and persistence under nutrient-limited conditions encountered during infection.

**Table 3:** Secretory systems specific proteins in *Burkholderia* spp

Type	Virulence factor	Alternate name	Species	Characteristics and functions	References
T1SS	TolC	Outer membrane protein/transporter	*B. pseudomallei*	Transports large proteins, toxins, and enzymes	(Thomas et al. 2014; Morgan et al. 2017)
	HylA	Hemolysin	*B. pseudomallei, B. cepacia*	Induces hemolysis, activates neutrophil degranulation markers	(Hutchison et al. 1998a)
	BicA	*Burkholderia* toxin	*B. pseudomallei, B. mallei, B. cepacia, B. cenocepacia*	Involved in intracellular replication in macrophages, cell-to-cell spread, and multinucleated giant cell formation	(Vellasamy et al. 2011; Stockton et al. 2023)
T2SS	ZmpA, ZmpB	Zinc metalloprotease	*B. cenocepacia*	Facilitates degradation of host proteins and modulates immune responses	(Holden et al. 2008)
	MprA	Metalloprotease A	*B. pseudomallei*	Elicit strong immune responses	(Chin et al. 2012)
	BMAA0553	Ser/Thr protein phosphatase (hypothetical protein)	*B. mallei*	Interacts with Rho GDP inhibitors for actin cytoskeletal rearrangement	(Memišević et al. 2013)
T3SS	BipB	*Burkholderia* invasion protein B/translocator protein BipB	*B. pseudomallei*	Enables invasion, MNGC formation, cell-to-cell spread, and apoptosis	(Suparak et al. 2005; Allwood et al. 2011)
	BipC	*Burkholderia* invasion protein C/effector protein BipC	*B. pseudomallei*	Interferes with host cytoskeleton, binds, and polymerizes actin	(Kang et al. 2016; Vander Broek and Stevens 2017)
	BipD	*Burkholderia* invasion protein D/needle tip protein	*B. pseudomallei*	Facilitates ubiquitination by binding to kelch-like proteins involved in E3 ubiquitin ligase complexes and to reduce mitochondrial ROS and induce mitophagy	(Nan et al. 2024)
	BopA	*Burkholderia* outer protein A	*B. pseudomallei*	BopA is said to bind to signalling proteins provoked by atg5, thereby escaping autophagic process. Helps survive pharmacologically induced autophagy	(Cullinane et al. 2008)
	BopB	*Burkholderia* outer protein B	*B. pseudomallei*	A GTP cyclohydrolase I co-regulated by BsaN with other T3SS-3 effector proteins	(Chen et al. 2014)
	BopC	*Burkholderia* outer protein C	*B. pseudomallei*	Helps in epithelial cell invasion and escape from phagocytic vesicles, promoting intracellular survival	(Srinon et al. 2013)
	BopE	Guanine nucleotide exchange factor BopE	*B. pseudomallei*	Invades non-phagocytic cells, induces actin rearrangement, and facilitates guanine exchange factor activity in ras-related proteins	(Stevens et al. 2003)
	BapA	*Burkholderia* effector protein A/beta alanine-specific aminopeptidase	*B. pseudomallei*	Helps the bacteria with its intracellular lifecycle; aids in cell attachment, entry, and cytoskeletal rearrangement	(Choh et al. 2021)
	BapB	*Burkholderia* effector protein A/Acyl carrier protein	*B. pseudomallei, B. mallei*	Negatively regulates BopE, which is involved in invasion and actin rearrangement	(Treerat et al. 2015)
	BicA	Bicarbonate transporter	*B. pseudomallei*	Activates proteins involved in inflammatory response modulation by regulating T3SS and T6SS	(Sanchez-Villamil et al. 2022)
	BicP	Type 3 secretion chaperone BicP	*B. pseudomallei, B. mallei, B. thailandensis*	A protein chaperone known to bind to BopA and helps stabilize it, thereby promoting autophagy escape	(Panina et al. 2005; Burtnick et al. 2008)
	BprB	Two-component system response regulator BprB	*B. pseudomallei, B. humptydooensis*	BprB is a regulator of T3SS-3 cluster and transcription and is involved in phosphorelay signal transduction system	(Sun et al. 2010)
	BprD	T3SS transcription regulator	*B.pseudomallei*	Negatively regulates BprC, which is essential for T6SS expression	(Chirakul et al. 2014)
	BsaN	Central regulator protein (T3SS-3)	*B. pseudomallei*	Regulates the expression of T3SS effectors, BprC, also facilitates expression of VirAG. VirAG and BprC expression are essential for T6SS1 expression inside host cells	(Chen et al. 2014)
	BsaQ	Inner membrane ring structural protein (T3SS-3 basal body component)	*B.pseudomallei, B. mallei, B.thailandensis, B. cepacia*	Impairment of phagosomal escape is seen in BsaQ mutant strain, decreasing intracellular survival. BopE and BipD secretion is also impaired in BsaQ mutants	(Muangsombut et al. 2008)
	BsaR	Putative T3SS-3 chaperone (likely SicA homolog)	*B. pseudomallei*	Computationally predicted to be a chaperone of BopE	(Vander Broek and Stevens 2017)
	BsaU		*B. pseudomallei, B. mallei, B. thailandensis*	It is essential for the proper assembly of T3SS-3 needle complex, which is facilitated by effector protein BopE and translocation tip protein BipD	(Pilatz et al. 2006)
	BsaZ	Secretion apparatus protein BsaZ	*B. pseudomallei, B. mallei, B. thailandensis, B. ubonensis, B. cepacia, B. mayonis*	Enables the escape of bacteria from the phagosome. It helps in the secretion of effector and translocation proteins	(Stevens et al. 2002)
	GvmR	Globally acting virulence and metabolism regulator	*B. pseudomallei*	Regulates the growth, biofilm formation, and motility of bacteria; Upregulates virulence factors like T3SS and T6SS effectors	(Duong et al. 2018)
	Chbp	Cyclomodulin Cif homolog	*B. pseudomallei*	This virulence factor performs deamidation of the Gln40 residue of ubiquitin and ubiquitin-like NEDD8, which impairs the process of ubiquitination. This further creates a stress in actin fiber formation and causes cell cycle arrest	(Cui et al. 2010)
	BMAA1865	T3SS effector (hypothetical protein)	*B. mallei*	Interacts with RhoGTPases for host entry and promotes actin cytoskeletal rearrangement	(Memišević et al. 2013)
	BPSS1517	Uncharacterized protein	*B. pseudomallei*	Chaperone protein which interacts with BopC	(Muangman et al. 2011)
T4SS	PilA	Type IV pilin protein (T4SS-associated pili subunit)	*B. pseudomallei*	Pili plays an important role in bacterial adhesion to host cells, which further contributes to infection of cells	(Essex-Lopresti et al. 2005)
T5SS	BpaC	T5SS (exact subclass often unclassified)	*B. pseudomallei*	Adhesion and virulence; exact mechanism under study	(Hatcher et al. 2015)
	BimA	Autotransporter	*B. pseudomallei, B. mallei*	Enhances actin-based mobility; this protein is virulent in *B. pseudomallei* and avirulent in *B.mallei*	(Lazar Adler et al. 2011)
	BoaA	Autotransporter (Hep_Hag family)	*B. pseudomallei*	Adheres to epithelial cells	(Lazar Adler et al. 2011)
	BoaB	Surface-exposed protein, autotransporter	*B.lata*	Likely involved in adhesion; exact function less characterized	(Lazar Adler et al. 2011)
T6SS	TssE	Type 6 secretion system basal plate subunit	*B. mallei, B. pseudomallei, B. cepacia*	MNGC formation, actin polymerization	(Burtnick et al. 2009)
	TssM		*B. pseudomalleiB. mallei, B. cenocepacia*	Functions as a deubiquitinase; downregulates innate immune response	(Burtnick et al. 2014)
	TssN		*B. pseudomallei*	Acts as a deubiquitinase and enhances phagosomal escape	(Memišević et al. 2013)
	PpiB	Peptidyl-prolyl cis–trans isomerase	*B. pseudomallei, B. cenocepacia, B. mallei*	Regulates efflux pumps, antibiotic resistance; forms MNGCs and biofilm; helps in protein folding	(Bzdyl et al. 2022)
	Hcp1	Hemolysin-coregulated protein 1	*B. mallei, B. pseudomallei, B. cenocepacia*	Binds to host cells for bacterial uptake, forms MNGCs and enables APC binding	(Burtnick et al. 2011)
	VgrG-5		*B. pseudomallei*	The C-terminal domain of this protein inserts inself into the host cell membrane and brings about fusion of cells. This helps the bacteria to spread from one cell to another	(Schwarz et al. 2014)
	BPSS1504	Cluster 1 T6SS secretion protein	*B. pseudomallei*	Mutation studies on this protein showed impaired MCGC formation and intracellular replication. It is also involved in the regulation of Hcp1 (hemolysin coregulated protein1)	(Hopf et al. 2014)

In *B. cepacia* complex species like *B. cenocepacia*, T1SS secretes cable pili-associated hemolysins capable of hemolyzing erythrocytes and neutrophils, which trigger phagocyte degranulation and apoptosis (Hutchison et al. 1998a). This enhances *B. cepacia’s* competitiveness in CF lungs by damaging host immune cells, indirectly inhibiting co-colonizers such as *Staphylococcus aureus* and *Pseudomonas aeruginosa* (Hutchison et al. 1998). Overall, the T1SS is vital for *Burkholderia* spp.’s ability to interact, adapt, and survive in the host as well as in varied environmental conditions. ABC transporter systems are known to function both independently and also as a part of T1SS, thereby influencing the accessibility and immunogenicity of *Burkholderia* spp. Thus, T1SS-associated secreted proteins, particularly surface-exposed and immunogenic substrates, may represent potential targets for antigen discovery, although their utility in vaccine development remains to be explored.

### Type 2 secretion system

**Fig. 3:**
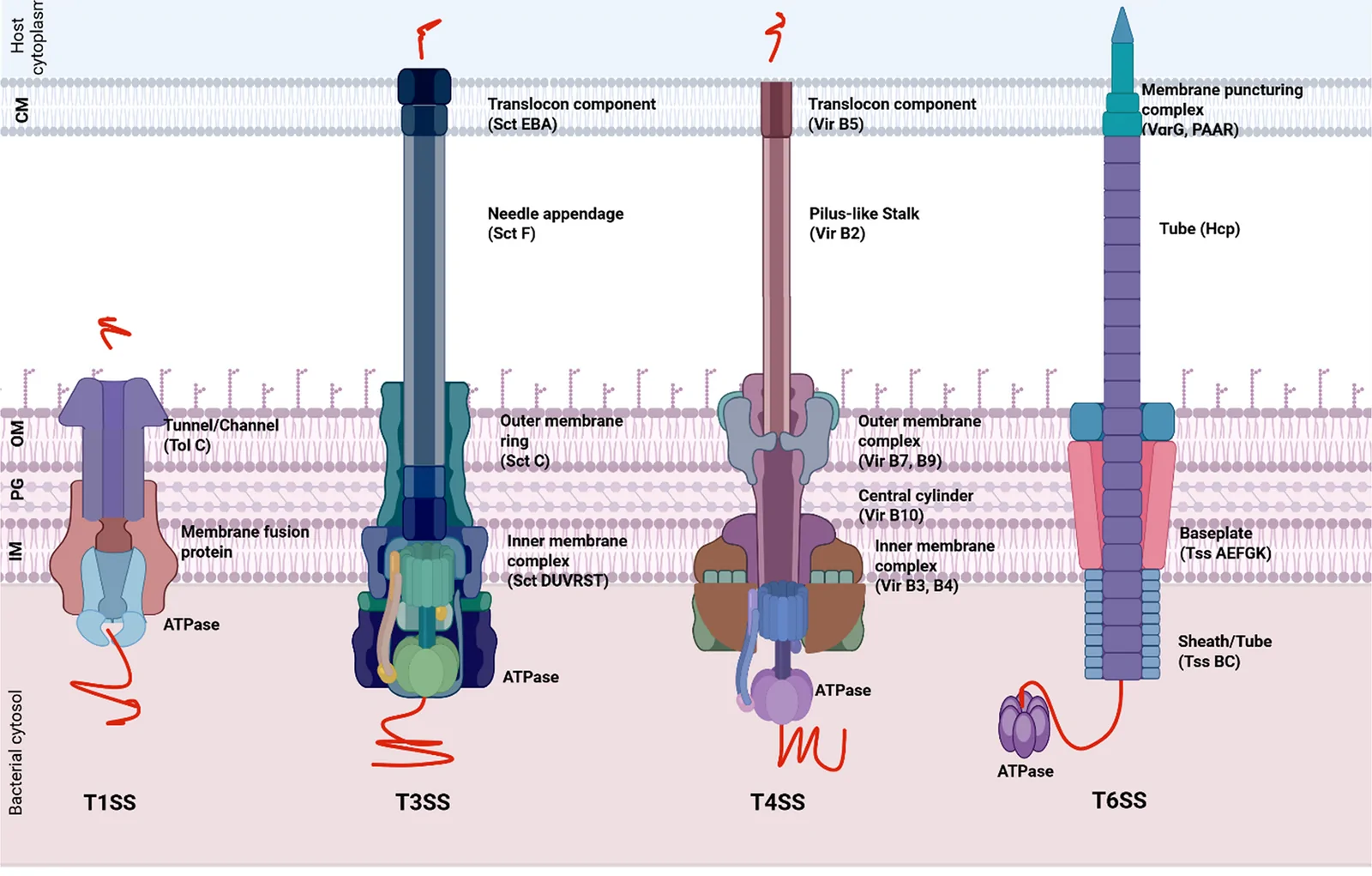
Schematic representation of the Type 1, 3, 4, and 6 secretion systems (T1SS, T3SS, T4SS, and T6SS).The key structural components of each secretion system have a two-membrane-spanning architecture. Essential elements involved in the assembly, substrate translocation, and interaction with host cells are depicted, emphasizing their role in pathogenicity and host adaptation

The Type 2 secretion system (T2SS) is a dual-step protein export pathway in Gram-negative bacteria, which enables the translocation of folded proteins from the periplasm across the outer membrane into the extracellular milieu (Figs. 2 and 3) (Shaliutina-Loginova et al. 2023). Proteins secreted via the T2SS conventionally have N-terminal signal peptides that are recognised by the secretory (Sec) or twin-arginine translocation (Tat) pathways, facilitating their transport across the inner membrane into the periplasm, where they fold into their active conformations (Shaliutina-Loginova et al. 2023). T2SS also plays a vital role in secreting enzymes, toxins, and other virulence-associated factors to enhance bacterial survival and induce pathogenesis. In addition, T2SS is considered the terminal branch of the general secretory pathway (Gsp) (Sandkvist 2001).

T2SS aids in the transport of these folded proteins from the periplasm to the exterior of the cell (Korotkov and Sandkvist 2019; Naskar et al. 2021). Structurally, it comprises four major components: (i) a cytoplasmic secretion ATPase that provides energy, (ii) an inner membrane platform that anchors the system and supports pseudopilus assembly, (iii) a periplasmic pseudopilus that functions as a molecular piston to propel substrates, and (iv) an outer membrane complex that forms the secretion pore (Korotkov and Sandkvist 2019; Naskar et al. 2021). T2SS is evolutionarily related to the Gram-negative bacteria’s Type IV pilus system, suggesting shared mechanisms underlying motility and protein secretion (Fehlner-Gardiner et al. 2002; Denise et al. 2019).

T2SS was first identified in *Klebsiella oxytoca*, where it promotes the release of starch-hydrolysing lipoprotein pullulanase (d’Enfert et al. 1987). In *B. pseudomallei*, T2SS consists of a minimum of 12 conserved Gsp proteins (Korotkov and Sandkvist 2019; Shaliutina-Loginova et al. 2023). The inner membrane platform stabilizes the complex and links it to the GspE ATPase, while the pseudopilus, primarily composed of GspG, drives protein extrusion through the outer membrane secretin GspD. Consistent with this mechanism, *B. pseudomallei* utilizes T2SS to secrete enzymes such as proteases, collagenases, lipases, and phospholipase C (DeShazer et al. 1999; Sandkvist 2001). Proteomic analyses have identified approximately 50 T2SS-associated substrates, which can be functionally grouped into roles in tissue degradation, immune modulation, and nutrient acquisition. Substrates involved in tissue degradation include collagenases; non-hemolytic phospholipase C (PlcN1, PlcN2); lipases; glycoside hydrolases; and pectinacetylesterase family proteins, which facilitate host tissue breakdown and bacterial dissemination. Those contributing to immune modulation and stress adaptation include Burkholderia lethal factor 1 (BLF1), superoxide dismutase (SodB), acid phosphatase (AcpA), PhoPQ-regulated proteins, and Ser/Thr phosphatases, which aid in immune evasion and protection against host defenses. In contrast, proteins associated with nutrient acquisition and metabolic adaptation include aminopeptidases, solute-binding proteins, levansucrase (SacB), cholesterol oxidase, and aldehyde oxidase, which support bacterial growth under nutrient-limited conditions. Among these, phospholipase C (PlcN1/PlcN2) and BLF1 are considered key virulence-associated effectors (Burtnick et al. 2014).

T2SS also promotes the secretion and export of extracellular toxins or enzymes that aid in infection via degradation of host barriers such as mucus and extracellular matrix components thereby weakening the host defenses. These effectors generally act extracellularly and do not directly enter the host cytosol (Korotkov and Sandkvist 2019). Effective host cell targeting often requires coordinated activity with other secretion systems, such as T3SS or T6SS, which directly inject effectors into host cells. In *B. cenocepacia*, T2SS exports zinc-dependent metalloproteases ZmpA and ZmpB, whereas T6SS mediates their translocation from the *B. cenocepacia*-containing vacuole into the host cytosol most likely via membrane disruption. These effectors activate the NLRP3/ASC inflammasome in the cytosol, leading to IL-1β secretion and pyroptotic cell death as seen in vitro conditions (Rosales-Reyes et al. 2012).

In *B. pseudomallei*, through genetic and functional studies, the validated immunogenic metalloprotease MprA secreted via T2SS has been identified as a potential vaccine candidate (Lee and Liu 2000; Valade et al. 2004). Recombinant MprA induces smBpF4-specific antibodies and elicits a Th2 immune response in immunized mice (Chin et al. 2012). Overall, T2SS plays a central role in bacterial-host interactions and significantly enhances pathogenic potential through efficient delivery of virulence factors (Table 3).

### Type 3 secretion system

The Type 3 secretion system (T3SS) is a specialized protein export apparatus employed by many Gram-negative bacteria to directly translocate effector proteins from the bacterial cytosol into host cells without a periplasmic intermediate. Structurally, T3SSs are evolutionarily related to the bacterial “flagellum” and function as a needle-like “injectisome,” acting as a molecular syringe to deliver virulence factors into host cells upon contact (Wallner 2021). Cryo-electron microscopy studies have revealed that T3SSs consist of an inner-membrane-anchored basal body and injectisome-sorting platform, a double-membrane-spanning export apparatus, a cytoplasmic ATPase that drives protein unfolding and secretion, and an extracellular needle-tip translocon complex that forms pores in host membranes (Figs. 2 and 3) (Bergeron and Marlovits 2022). Although T3SS and bacterial flagella share a common ancestry, the flagellum by itself is not a functional part of T3SS. The needle tip can sense contact with host cells and undergoes conformational changes that would trigger/regulate the effector protein secretion through classical signal transduction mechanisms (Lara-Tejero and Galán 2019). Effector secretion through the T3SS follows a hierarchical order, with early secretion of translocon components to establish host membrane pores, followed by delivery of effector proteins, which are recognized by short N-terminal secretion signals and often stabilized by dedicated chaperones prior to export thereby contributing to bacterial pathogenicity and intracellular survival (Lara-Tejero and Galán 2019; Worrall et al. 2023).

### Clusters of T3SS in *Burkholderia* spp.

Members of the *Burkholderia pseudomallei* group, viz., *B. pseudomallei*, *B. mallei*, and *B. thailandensis* encode three distinct T3SS clusters, namely T3SS-1, T3SS-2, and T3SS-3 (Rainbow et al. 2002; Vander Broek and Stevens 2017; Bzdyl et al. 2022). T3SSs are classified into multiple families based on the homology of their core structural genes, including the Ysc family (Ysc, Psc, Lsc, Asc, Vsc, Dsc, Bsc); the Inv-Mxi-Spa family; the Ssa-Esc family (SPI-2-encoded-Esc T3SS); the Hrc-Hrp 1 and Hrc-Hrp 2 families; the Rhizobiales family; and the Chlamydiales family (Troisfontaines and Cornelis 2005). T3SS-1 and T3SS-2 are evolutionarily related to plant-associated Hrc-Hrp systems, with T3SS-1 showing homology to Hrc-Hrp2 and T3SS-2 aligning with Hrc-Hrp1 families commonly found in phytopathogens (Rainbow et al. 2002; Stevens et al. 2002). In contrast, T3SS-3 belongs to the Inv-Mxi-Spa family, which is conserved among animal pathogens such as *Salmonella* sp. and *Shigella* sp., and plays a dominant role in mammalian infection (Rainbow et al. 2002; Stevens et al. 2002). The coexistence of both plant-associated and animal-associated T3SSs within the *B. pseudomallei* complex suggests an evolutionary strategy supporting environmental persistence, enabling survival across diverse ecological niches including soil, plants, protozoa, and mammalian hosts (Attree and Attree 2001).

Although T3SS-1 and T3SS-2 are not considered essential for mammalian virulence, emerging evidence indicates that these systems may still influence infection outcomes. A mutation in bpscN, encoding the ATPase of T3SS-1, resulted in reduced virulence in murine respiratory melioidosis models (Vander Broek and Stevens 2017) and decreased intracellular survival in RAW264.7 macrophages, while also increasing colocalization with the autophagy marker LC3 (D’Cruze et al. 2011). Despite this, the mutant preserved its ability to escape from phagosomes, suggesting a possible role of the *bpscN* gene product in *B. pseudomallei* pathogenesis and intracellular survival during murine infection (D’Cruze et al. 2011). The evolutionary similarity between plant pathogens such as *Pseudomonas syringae and* *Xanthomonas* species, which utilize Hrc-Hrp T3SSs to suppress host cell death in plants, and *B. pseudomallei* suggests that plant-associated T3SSs may represent ancestral virulence mechanisms adapted for multiple host types (D’Cruze et al. 2011).

### T3SS-3 in *Burkholderia* spp. virulence

Animal-associated T3SS-3 is the primary virulence-associated secretion system in *B. pseudomallei* and is essential for intracellular survival, phagosomal escape, and actin-based motility, since both processes are impaired in T3SS-3-deficient mutants through genetic and in vitro study (Stevens et al. 2002). Components of T3SS-3 are collectively referred to as the *Burkholderia* secretion apparatus (Bsa) and include structural proteins forming the injectisome (e.g., BsaL, BsaQ, BsaO, BsaP, BsaS, BsaV, BsaU, BsaT, BsaZ, BsaY); translocator proteins (BipB, BipC, BipD); and secreted effectors, including experimentally validated effectors such as BopA and BopE, as well as putative or less-characterized effectors (e.g., BopB, BopC, BapA and BapC) (Allwood et al. 2011; Vander Broek and Stevens 2017). The ATPase BsaS is critical for T3SS-3 function, driving effector unfolding and secretion, and bsaS mutants exhibit impaired phagosomal escape, reduced intracellular replication, and enhanced targeting by autophagy pathways in macrophages in an in vivo model (Gong et al. 2011) (Table 3). This is an evolutionarily conserved virulence strategy seen in *Listeria monocytogenes* (O toxin and ActA protein) and *Shigella flexneri* (LcsA) (Gong et al. 2011).

Among T3SS-3 effectors, experimentally validated effector BopA promotes phagosomal escape and intracellular survival by interfering with LC3-associated phagocytosis (LAP), a non-canonical autophagy pathway critical for antibacterial defense (Gong et al. 2011). Experimentally validated effector BopE functions as a guanine nucleotide exchange factor (GEF) that induces actin cytoskeleton rearrangements to facilitate bacterial invasion of epithelial and phagocytic cells (Figs. 2 and 3) (Muangman et al. 2011; Vander Broek et al. 2015).

BopC contributes to efficient vacuolar escape and epithelial cell invasion, as bopC mutants show delayed escape from J774A.1 macrophages and impaired penetration into A549 alveolar epithelial cells (Cullinane et al. 2008; Muangman et al. 2011). The translocator BipC plays a central role in host actin modulation and MAPK signaling, influencing the expression of over 1000 host genes associated with invasion and intracellular survival (Kang et al. 2016). *Burkholderia* invasion protein B (BipB) and *Burkholderia* secretion apparatus Q (BsaQ) help in bacterial invasion, evidenced by *bipB* and *bsaQ* mutants of *B. pseudomallei* showing defective penetration into HFF-1 skin fibroblasts (Kaewpan et al. 2022).

A recently characterized T3SS-3 effector, CHBP (cycle-inhibiting factor; Cif), induces host cell cycle arrest by blocking the G1-to-S phase transition. In neuronal SH-SY5Y cells, cif mutants exhibit increased actin-tail formation and multinucleated giant cell (MNGC) formation, implicating CHBP in the pathogenesis of neurological melioidosis (Rungruengkitkun et al. 2022). This highlights a specialized role for T3SS-3 effectors in tissue-specific disease manifestations.

In addition to its role in intracellular survival, T3SS-3 has been linked to host innate immune responses during *Burkholderia* spp. infection. Studies have shown that *Burkholderia* spp. induce inflammasome activation, leading to caspase-1-dependent maturation of pro-inflammatory cytokines such as IL-1β and IL-18, as well as pyroptotic cell death in macrophages (Ceballos-Olvera et al. 2011). While multiple bacterial factors contribute to this response, T3SS-mediated host-pathogen interactions are considered important in modulating these innate immune pathways (Tan et al. 2010). This highlights the dual role of T3SS in both promoting bacterial survival and influencing host inflammatory responses.

Collectively, T3SSs, particularly T3SS-3, are central to the pathogenic strategy of *Burkholderia* spp., enabling intracellular survival through coordinated effector delivery, manipulation of host cytoskeletal dynamics, evasion of autophagy and LAP pathways, and modulation of host cell cycle progression. Small-molecule T3SS secretion inhibitors have been reviewed as promising anti-virulence agents (Tang et al. 2024). While plant-associated T3SSs likely contribute to environmental fitness, T3SS-3 represents the dominant virulence determinant during mammalian infection, making it a critical focus for therapeutic and vaccine development.

### Type 4 secretory system

The Type 4 secretion system (T4SS) is a one-step translocation apparatus that mediates the direct transfer of protein and nucleoprotein substrates across the bacterial cell envelope into recipient cells without a periplasmic intermediate. T4SSs are evolutionarily related to bacterial conjugation systems and form double-membrane-spanning complexes equipped with surface pili that facilitate cell-to-cell contact and substrate delivery (Figs. 2 and 3) (Costa et al. 2023). Canonical Gram-negative T4SSs consist of approximately 12 conserved components (VirB1–VirB11 and VirD4) organized into functional modules, including an inner membrane complex, a conjugative pilus, and an outer membrane core complex (OMCC) bridging the inner and outer membranes (Costa et al. 2023). T4SSs can transfer proteins and DNA (nucleoproteins), aiding conjugative transfer of DNA, uptake and release of the genetic material, and translocation of effector proteins or DNA-protein complexes directly into recipient cells (Dugelay et al. 2025).

Recent cryo-electron microscopy studies of conjugative T4SSs, such as the plasmid R388 system, have resolved near-atomic structures and used co-evolution analysis to predict protein interactions and model the complete complex. The structural components of the T4SS include the inner membrane complex (IMC) made of VirB3, VirB4, and the N-terminal tails of VirB8; the arches consisting of VirB8 periplasmic domains; the stalk formed by pentamers of VirB5 and VirB6; and the outer membrane core complex (OMCC) composed of VirB7, VirB9, and VirB10. Additional supporting structures include a conjugative pilus, essential for DNA transfer, and three ATPases (Macé and Waksman 2024). Although the extent of effector secretion varies between species and ecological niches, T4SSs functionally mediate horizontal gene transfer, plasmid mobilization, and effector translocation, contributing to bacterial adaptation, virulence, and host interactions, (Macé and Waksman 2024).

Among *Burkholderia* spp., functional T4SSs (Table 3) have been most extensively characterized in *Burkholderia cenocepacia*, a member of the *Burkholderia cepacia* complex (Bcc). Two distinct T4SSs have been identified: a chromosomally-encoded VirB/D4 system involved primarily in plasmid mobilization and a plasmid-encoded Ptw (plant-tissue water-soaking) T4SS originally linked to plant pathogenicity (Twombly 2005). The Ptw T4SS is a chimeric system containing VirB/D4-like components and is required for water-soaking phenotypes in plant tissue, as disruption of key genes such as *ptwD4* abolishes this activity (Engledow et al. 2004). Beyond plant interactions, the Ptw T4SS contributes to *B. cenocepacia* intracellular survival in epithelial cells and macrophages. Mutants lacking functional T4SS display increased trafficking to degradative compartments and reduced persistence, suggesting a role in modulating phagosome maturation (Engledow et al. 2004). In contrast, the chromosomal VirB/D4 system appears structurally conserved but has not been conclusively linked to effector translocation during host infection, and its contribution is thought to be limited to conjugative DNA transfer (Zhang et al. 2009).

Notably, specific T4SS effector proteins have not yet been experimentally identified in *Burkholderia*, representing a major gap in understanding T4SS-mediated host modulation. Machine-learning-based effector prediction, particularly for under-characterized SSs, can accelerate effector discovery and fill current knowledge gaps (Tang et al. 2024). Beyond diagnostics and vaccines, targeting conserved secretion system ATPases, specifically in T3SS (e.g., SsaN, EscN), T2SS (e.g., GspE), and T4SS (e.g., VirB11), with small-molecule inhibitors offers a strategy to “disarm” pathogenic bacteria without directly killing them, potentially reducing selective pressure for resistance. Although much work on ATPase inhibitors has been conducted in other pathogens, small-molecule inhibition of secretion system ATPases remains an active area of interest in combating secretion-dependent intracellular invasion and virulence.

### T4SS distribution in other *Burkholderia* spp.

Genomic and experimental evidence indicates that members of the *B. pseudomallei* group, including *B. pseudomallei*, *B. mallei*, *B. thailandensis*, and *B. oklahomensis*, do not encode functional T4SSs, distinguishing them from Bcc members and plant-associated *Burkholderia* spp. (Angus et al. 2014). Environmental and endophytic *Burkholderia* strains may harbor plasmid-borne T4SSs that facilitate horizontal gene transfer and plant colonization, although mechanistic and functional validation remains limited (Jia and Lu 2024).

While T4SSs in other pathogens such as *Legionella* and *Brucella* are well-established effector delivery systems, *Burkholderia* spp. T4SSs remain poorly characterized at the effector level, limiting mechanistic insights into host manipulation (Dugelay et al. 2025).

Future studies integrating comparative genomics, machine-learning-based effector prediction, and functional validation are essential to uncover T4SS substrates and clarify their roles in *Burkholderia* spp. pathogenesis and environmental adaptation (Li et al. 2024).

### Type 5 secretory system

**Fig. 4:**
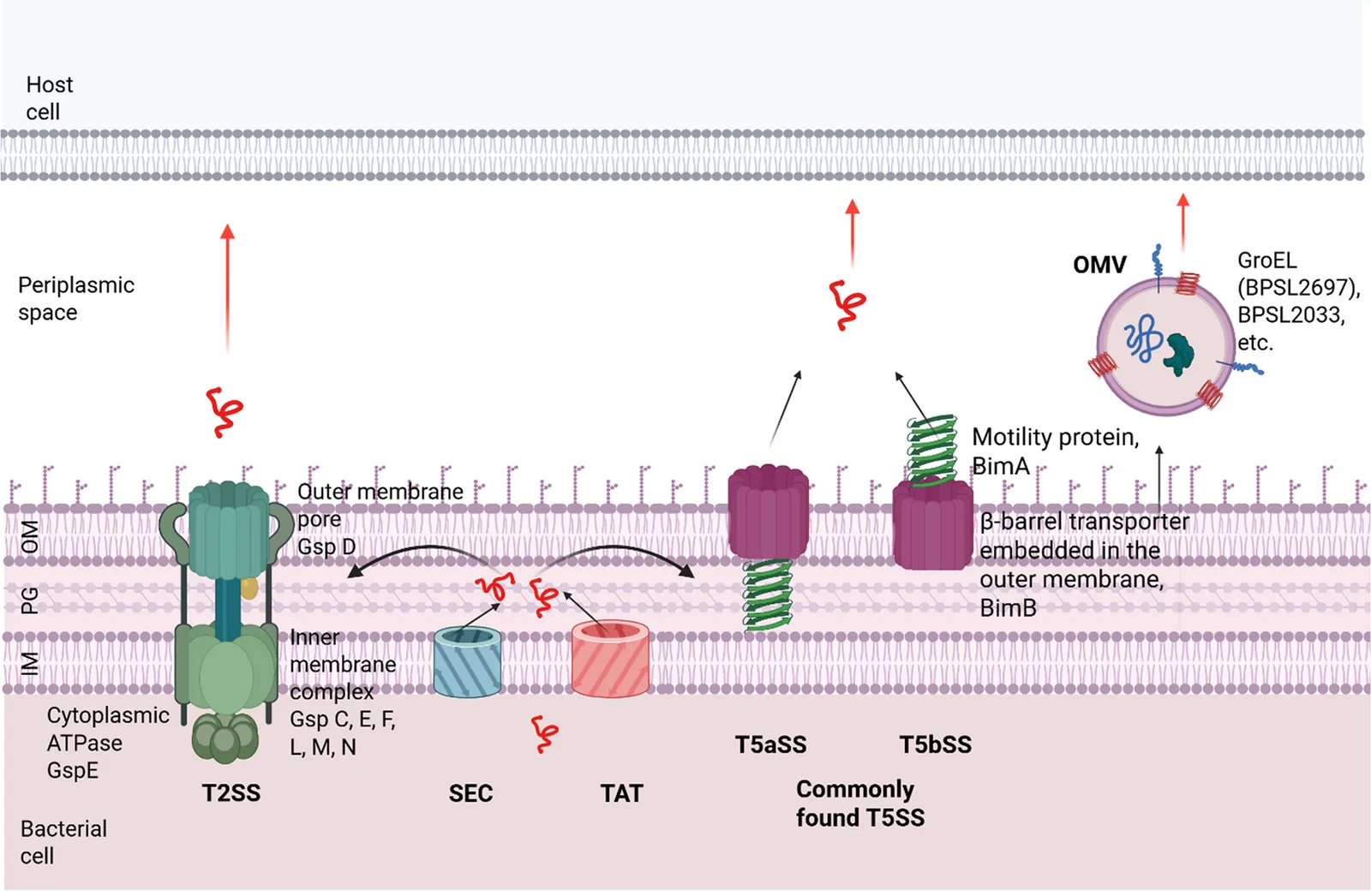
Schematic representation of the Type 2 and 5 secretion systems (T2SS, T5SS) and outer-membrane vesicle (OMV). The key structural components of each secretion system have single-membrane secretion architecture. Essential elements involved in the assembly, substrate translocation, and interaction with host cells are depicted, emphasizing their role in pathogenicity and host adaptation

The Type 5 secretion system (T5SS) is a dual step translocation system, delivering the effector proteins across the outer membrane (Figs. 2 and 4). T5SS is primarily responsible for the secretion of autotransporters. Within this system, autotransporters are transmembrane proteins composed of three main domains: (i) an N-terminal signal sequence directing the protein to the outer membrane, (ii) a passenger domain that is transported outside the cell to perform its function, and (iii) a C-terminal beta-barrel domain that embeds the protein in the outer membrane, acting as an anchor (Dautin 2021).

Initially, the protein is transported across the inner membrane via the Sec pathway utilizing its N-terminal signal peptide. Once in the periplasm, the protein undergoes a conformational change by allowing the C-terminal beta-barrel domain to insert into the outer membrane and thereby forming a pore in the outer membrane. The extracellular passenger domain is subsequently translocated through this pore to the cell surface, where it performs its biological function. Finally, the beta-barrel domain remains anchored to the outer membrane, serving as the structural channel for the secretion process (Stevens et al. 2005; Nicolay et al. 2015). This secretion does not require a multi-protein apparatus, as the autotransporter itself facilitates both translocation and secretion.

In *Burkholderia* spp., autotransporter proteins play significant roles in virulence. Most of the autotransporters characterized in *B. pseudomallei* and *B. mallei* are trimeric with three identical subunits, such as BpaA, BpaB, BpaC, which are involved in adhesion and invasion (Stevens et al. 2005; Lazar Adler et al. 2011; Garcia et al. 2016). A notable exception is BimA, an autotransporter that promotes actin polymerization and intracellular motility, thereby facilitating the bacterial spread between host cells. Comparative sequence analysis of the passenger domains of BimA from *B. pseudomallei, B. mallei*, and *B. thailandensis* indicates they polymerize host actin through different mechanisms. The actin-binding regions of BimA in *B. thailandensis* include both the WASP homology 2 (WH2) and central and acidic motifs (CA), which are crucial for the activation of the Arp2/3 complex to modulate the host cytoskeleton via facilitating actin polymerization. These motifs can help the protein interact with actin inside the host cells, promoting intracellular motility. In contrast, BimA of *B. pseudomallei* and *B. mallei* have lowered WH2 motifs and lack the CA region (Cagape et al. 2024). BimA in *B. pseudomallei* is known to be crucial for actin binding and nucleation; the exact mechanism of actin nucleation is still unknown. These species-specific variations in BimA lead to variations in actin tail formation with distinct filament formation, thereby influencing the bacterial mobility and ability to fuse with the host cells. This may partly explain the reason behind the species *B. pseudomallei* and *B. mallei* being more virulent than *B. thailandensis* (Benanti et al. 2015; Cagape et al. 2024). Classical transporters BcaA and BcaB (Campos et al. 2013; Lafontaine et al. 2014), along with BatA (Lafontaine et al. 2019) are known for their importance in processes such as host cell invasion and intracellular growth. Moreover, these can also serve as promising targets for developing vaccines or immunotherapies to counteract the *Burkholderia* spp. infections.

### Type 6 secretory system

The Type 6 secretion system (T6SS) is a single-step, contractile nanomachine that delivers effector proteins into host or competitor cells. Structurally, it resembles an inverted bacteriophage tail and functions through a sheath-tube apparatus which punctures the target cell membrane (Figs. 2 and 3) (Cherrak et al. 2018). T6SS comprises 20 core proteins related to phage tail components (Cherrak et al. 2018), including a contractile sheath (TssB, TssC) wrapped around an inner tube (Hcp), spike complex proteins (VgrG, PAAR) (Shneider et al. 2013), and a baseplate (TssE, TssF, TssG, TssK) linked to a membrane complex (TssJ/TssL/TssM) (Brunet et al. 2015; Rapisarda et al. 2019). The contracted sheath is recycled by the ATPase ClpV (TssH) for subsequent firing cycles (Pietrosiuk et al. 2011; Lennings et al. 2019).

T6SSs have diversified into six different clusters (T6SS-1 through T6SS-6) based on core gene variation and effector repertoires, with specific clusters associated with different functions such as interbacterial competition, metal ion acquisition, and intracellular life-cycle modulation (Shalom et al. 2007; Nguyen et al. 2018; Lennings et al. 2019). *B. pseudomallei* expresses all six of the types, while *B. thailandensis* expresses five of the types and several have distinct roles in ecology and pathogenesis: T6SS-1 is implicated in interbacterial competition and stress adaptation, T6SS-2 in manganese and zinc homeostasis, and T6SS-5, also called T6SS-1 in older literature, in intracellular spread and host cell manipulation (Nguyen et al. 2018; Lennings et al. 2019).

### Regulatory mechanisms of T6SS-1

T6SS expression is tightly regulated by environmental cues and signal transduction systems, including quorum sensing and the VirAG two-component system in *B. pseudomallei* (Burtnick and Brett 2013), which directly activates genes such as well-characterized bimA, vgrG, hcp1, tssA, and tssM, all of which are essential for T6SS assembly (Chen et al. 2011). Cross-regulation by the T3SS-3 regulator BsaN modulates T6SS transcripts, emphasizing inter-system coordination during infection (Lennings et al. 2019). Iron limitation and other nutrient stresses also influence T6SS-1 expression, reflecting a role in survival under intracellular stress conditions (Lennings et al. 2019). *B. pseudomallei* can effectively evade the immune responses, and studies also show that the expression of the T6SS-1 gene is inversely related to iron availability, suggesting that nutrient limitation, such as iron, can influence or regulate the T6SS-1 activity (Burtnick and Brett 2013). While Nramp1 is known to reduce intracellular iron availability to limit bacterial growth, *B. pseudomallei* evades this effect and does not stimulate the pro-inflammatory cytokines such as TNF-α, IL-1β, and IL-6, while sustaining IL-10 production (Muangsombut et al. 2017).

T6SS-2 contributes to contact-independent export of manganese- and zinc-binding effectors (TseM and TseZ, respectively), supporting redox homeostasis under oxidative stress via interactions with TonB-dependent transporters (BhuR and MnoT), and is activated by ROS and GvmR, with repression by regulators such as OxyR, TctR, and Zur (DeShazer 2019). TctR, a transcriptional regulator of the MarR family, negatively regulates seven gene clusters, including T6SS-2, while activating three others (Losada et al. 2018). These roles highlight the broader functional scope of T6SSs beyond direct host targeting.

### Cluster T6SS-5 in *Burkholderia* spp. virulence

Among *Burkholderia* T6SS clusters, T6SS-5 plays a key role in virulence, facilitating intracellular spread via multinucleated giant cell (MNGC) formation and host membrane disruption under conditions mimicking immune cell contact (Plum et al. 2024). Disruption of T6SS-5 in *B. thailandensis* severely attenuates virulence in wild-type mice but not in MyD88-deficient animals, highlighting its role in overcoming innate immune defenses during melioidosis-like infection models (Lennings et al. 2019). Specific proteins within the T6SS-5 cluster, including VgrG5 C-terminal domains, are critical for membrane fusion and intercellular spread, while Hcp proteins serve as both structural tubes and chaperones for effector loading (Lennings et al. 2019), key molecular features, biological functions, and evidence types supporting the role of individual T6SS components are summarized in Table 4. *B. pseudomallei* uses its T6SS-5 to induce host cell fusion, enabling intercellular spread while avoiding extracellular immune defenses. Interestingly, T6SS-5 driven fusion activates the cGAS-STING pathway through micronuclei formation, triggering type I IFN gene transcription without actual IFN protein production and instead promoting autophagic cell death. These findings suggest that the cGAS-STING pathway senses abnormal cell fusion as a danger signal, limiting aberrant cell division and preventing potential transformation by inducing programmed autophagic death (Ku et al. 2020).

**Table 4:** Key type VI secretion system (T6SS) clusters and components in *Burkholderia* species, highlighting molecular players, functional roles in intracellular dissemination and virulence, associated immune or cellular phenotypes, and representative studies

T6SS cluster/component	Species	Key molecular players	Functional role	Immune/cellular phenotype	Key references
T6SS-5	*B. thailandensis*	VgrG5, Hcp5, Tss proteins	Intercellular spread via membrane protrusion lysis	Reduced recruitment of galectin-3, LC3, LAMP1; immune evasion	(Plum et al. 2024)
T6SS-5	*B. pseudomallei*, *B. thailandensis*	VgrG (C-terminal domain), tssH-5, tssI-5, tssM-5	MNGC formation and intracellular dissemination	Enhanced intracellular spread and virulence	(Shalom et al. 2007)
VgrG5 (T6SS-5)	*B. pseudomallei*	C-terminal hydrophobic domains	Fusogenic activity and membrane fusion	Polar localization; MNGC formation	(Toesca et al. 2014)
Hcp (core T6SS tube)	*B. pseudomallei*	Hcp (hexameric ring)	Structural tube; effector chaperone	Required for effector delivery	(Silverman et al. 2013)
Hcp (pan-T6SS)	*B. pseudomallei*	Hcp (all six T6SSs)	Immunogenic protein; vaccine candidate	Strong antibody responses	(Burtnick et al. 2009)
Hcp1 (T6SS-1)	*Burkholderia*, *Pseudomonas*	Hcp1	Intracellular growth and cytotoxicity In vitro (RAW264.7 macrophages)	APC binding; antibody induction; virulence attenuation upon deletion	(Burtnick et al. 2009)
Hcp isoforms (Hcp2, Hcp6)	*B. pseudomallei*	Hcp2, Hcp6	Functional diversification	Hcp6 constitutive activity; Hcp2 ~ 80% protection against lethal infection	(Burtnick et al. 2011)
T6SS-1 regulation	*B. pseudomallei*	VirAG, media composition	Environmental regulation of virulence	Enhanced survival in host-like conditions	(Burtnick and Brett 2013)
T6SS-5 genes (diagnostic)	*B. pseudomallei*	tssA-5, tssB-5, tssC-5, tagD-5, tssF-5, hcp-5, vgrG-5	Molecular detection and surveillance	High-sensitivity detection	(Semail et al. 2022)
Antibiotic modulation of T6SS	*B. pseudomallei*	Hcp2, T6SS-3/4/6 genes	Antibiotic-responsive regulation	Enhanced transcription upon ciprofloxacin	(Losada et al. 2018; Semail et al. 2022)

### Integrated model of secretion system coordination in *Burkholderia* spp. pathogenesis

**Fig. 5:**
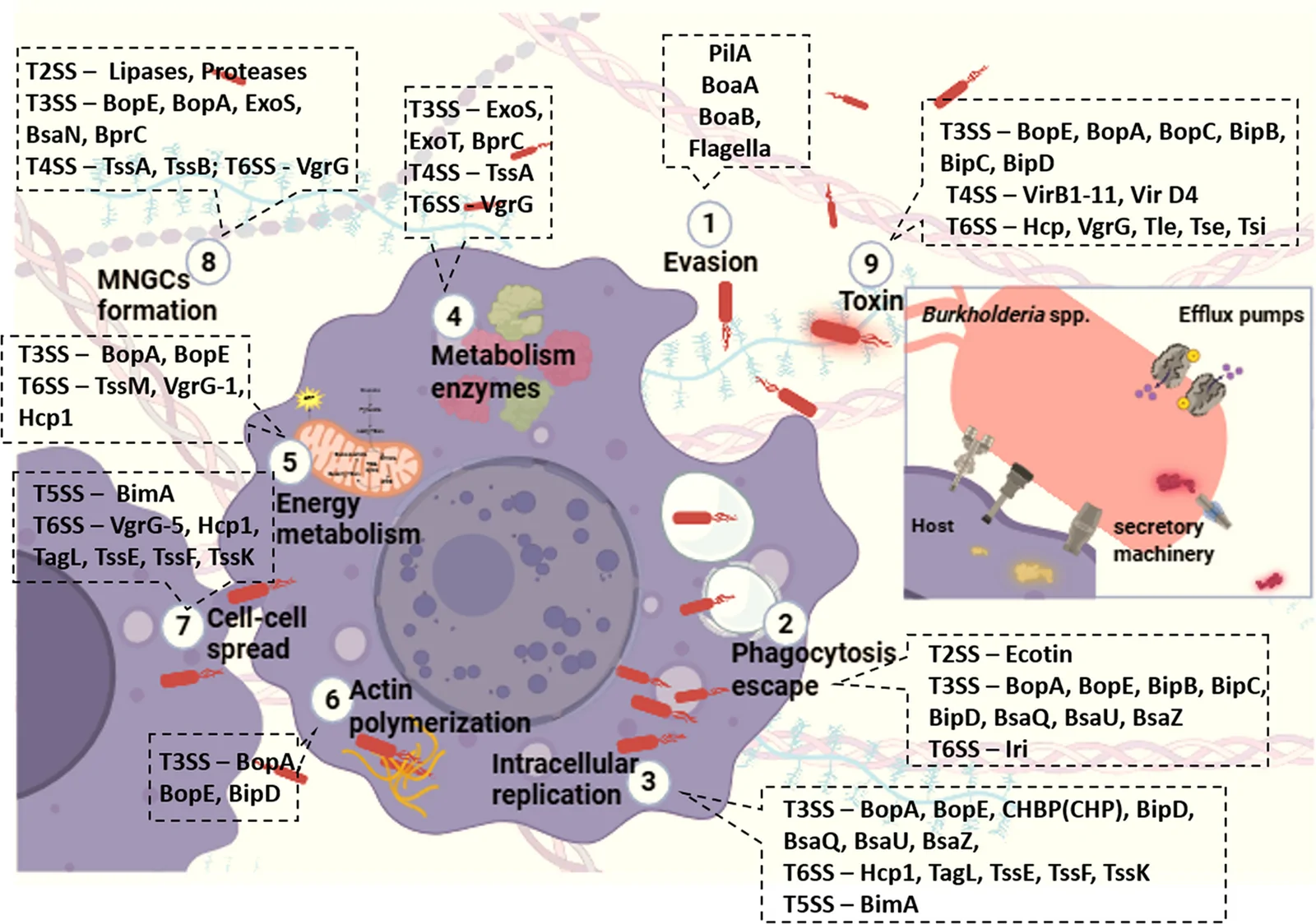
Stages of *Burkholderia* spp. pathogenicity mainly mediated by Type 2, 3, 4, 5 and 6 secretion systems. The schematic illustrates a stage-wise model of infection in which multiple secretion systems contribute in a coordinated and partially overlapping manner to host invasion, intracellular adaptation, and interbacterial interactions. During stage 1 (Evasion), surface-associated factors including PilA, BoaA, BoaB, and flagella mediate initial host interaction and contribute to immune evasion. This is followed by stage 2 (Phagocytosis escape), where T2SS-secreted Ecotin, T3SS effectors (BopA, BopE, BipB, BipC, BipD, BsaQ, BsaU, BsaZ), and the T6SS-associated factor Iri are involved in facilitating escape from phagosomal compartments. In stage 3 (Intracellular replication), T3SS effectors (BopA, BopE, CHBP/CHP, BipD, BsaQ, BsaU, BsaZ) along with T6SS components (Hcp1, TagL, TssE, TssF, TssK), BimA (T5SS) support bacterial persistence within the host cell. This intracellular phase extends into stage 4 (Metabolism enzymes), where T3SS-associated proteins (ExoS, ExoT, BprC), together with T4SS (TssA) and T6SS (VgrG), contribute to metabolic adaptation and host cell modulation, and into stage 5 (Energy metabolism), which includes the T3SS effector BopA BopE and T6SS components (TssM, VgrG-1 and Hcp1) supporting intracellular maintenance and energy-associated processes. Progressing further, stage 6 (Actin polymerization) involves T3SS effectors (BopA, BopE, BipD) associated with actin cytoskeletal rearrangements, which transitions into stage 7 (cell-cell spread), where T5SS (BimA) and T6SS components (VgrG-5, Hcp1, TagL, TssE, TssF, TssK) contribute to intercellular dissemination. In stage 8 (Multinucleated giant cell formation, MNGCs), T3SS effectors (BopA, BopE, ExoS, BsaN, BprC), along with T4SS (TssA, TssB) and T6SS components (VgrG) are associated with host cell fusion processes. Finally, stage 9 (Toxin secretion and interbacterial interactions) involves T3SS (BopE, BopA, BopC, BipB, BipC, BipD), T4SS (VirB1-11, VirD4), and T6SS effectors (Hcp, VgrG, Tle, Tse, Tsi), highlighting their roles in toxin delivery and bacterial competition, with efflux pumps and secretory machinery further contributing to bacterial survival. Collectively, these systems act in an overlapping and coordinated manner, allowing *Burkholderia* spp. to invade host cells, survive intracellularly, evade immune responses, and outcompete microbial rivals

During *Burkholderia* spp. infection, multiple secretion systems operate in a coordinated and partially overlapping manner to support host invasion, intracellular survival, and persistence (Fig. 5). Collectively, and as illustrated in Fig. 5, these systems form an interconnected network rather than acting as independent modules, with temporal and functional overlap enabling *Burkholderia* spp. to efficiently transition between extracellular survival, intracellular replication, immune evasion, and competitive persistence within complex host environments.

Initial host immunity evasion (stage 1) is mediated by surface-associated factors such as PilA, BoaA, BoaB, and flagella, along with secreted substrates, with T1SS contributing to the export of RTX toxins that promote host cell damage, immune modulation, and nutrient acquisition (Harland et al. 2007; Pourhassan N et al. 2022; How et al. 2022). Following host entry, T3SS-3 assumes a central role by enabling phagosomal escape (stage 2) and intracellular survival through the delivery of effectors such as BopA, BopE, and Bip proteins, which manipulate host cytoskeletal dynamics, autophagy pathways, and cellular signaling (Stevens et al. 2002; Gong et al. 2011; Muangman et al. 2011; Vander Broek et al. 2015). In parallel, T2SS supports these processes through the secretion of degradative enzymes, including proteases, lipases, ecotin and phospholipases, which act extracellularly or within vacuolar compartments to promote tissue disruption and nutrient acquisition, thereby functionally complementing T3SS-mediated intracellular activities (DeShazer et al. 1999; Burtnick et al. 2014; Korotkov and Sandkvist 2019). As infection progresses (stage 3 and beyond), experimentally validated components such as Hcp1 mediate host cell fusion, cytosolic spread, and modulation of immune signaling pathways in vitro, in vivo, and genetic studies, alongside associated or less-characterized effectors (e.g., VgrG proteins) that contribute to interbacterial competition (Nguyen et al. 2018; Lennings et al. 2019; Plum et al. 2024). This intracellular phase is subsequently enhanced during actin polymerization and dissemination (stages 6–7) by T5SS autotransporters such as BimA, which drive actin-based motility and facilitate direct cell-to-cell spread, enabling bacterial dissemination without extracellular exposure (Benanti et al. 2015; Cagape et al. 2024). In contrast, T4SSs, primarily described in *Burkholderia cepacia* complex species, are more closely associated with horizontal gene transfer and intracellular adaptation, although their role in direct effector translocation during host infection remains less clearly defined (Engledow et al. 2004; Zhang et al. 2009). In addition, outer membrane vesicles (OMVs) function as auxiliary delivery systems that transport toxins, enzymes, and virulence-associated factors, extending the functional reach of secretion systems beyond direct cell contact and further enhancing host-pathogen interactions and interbacterial competition (Kulp and Kuehn 2010; Baker et al. 2021).

### Bacterial outer membrane vesicles

Bacterial outer membrane vesicles (OMVs) are nanoscale, spherical structures (approximately 20–250 nm) released from the outer membrane of Gram-negative bacteria through membrane budding (Fig. 4) (Huang et al. 2022). Although OMVs are not classified as secretion systems, they function as supportive secretion vehicles, enabling the extracellular transport of nucleic acids, lipids, lipoproteins, toxins, and virulence-associated proteins. OMVs interact with the host cells through surface binding, endocytosis or membrane fusion, promoting immune modulation, host cell manipulation and bacterial pathogenesis (Jones et al. 2002; Baker et al. 2017). Recent studies have indicated that OMVs can act as supportive delivery systems for effectors that typically require the cell-to-cell contact mediated by T3SS/T4SS, thereby extending the reach of these effectors beyond direct bacterial-host interactions (Baker et al. 2021; Ketterer et al. 2026). OMVs can be actively recruited by T6SS effectors to enhance bacterial competition and facilitate horizontal gene transfer. For example, in *Pseudomonas* sp. and other Gram-negative bacteria, a T6SS-secreted LPS-binding effector captures OMVs containing antibacterial molecules or genetic material, effectively expanding the functional reach of T6SS beyond direct cell-to-cell contact (Baker et al. 2021; Li et al. 2022). In *Burkholderia* spp., OMVs play a vital role in virulence and microbial competition. *B. cepacia* OMVs contain proteins derived from various bacterial compartments thus inducing strong pro-inflammatory responses in vitro (Kim et al. 2020)*.* In addition, OMVs from *Burkholderia* spp. transport bioactives such as rhamnolipids and quinoline derivatives, including HMNQ that exhibits potent antibacterial activity against multidrug-resistant pathogens like *Acinetobacter baumannii*, thereby highlighting their role in interbacterial competition and antimicrobial defense (Wang et al. 2020). OMVs have also emerged as promising vaccine platforms. While whole-cell and live-attenuated *B. pseudomallei* vaccines demonstrate high immunogenicity in vivo models, safety concerns limit their clinical applicability. In contrast, OMV-based vaccines have shown protective efficacy in preclinical models, including macaques, preventing lung pathology and eliciting antibody-mediated protection with reduced inflammatory risk (Table 5) (Baker et al. 2021; Maphosa et al. 2023; Baker et al. 2025). While OMVs do not directly participate in the mechanistic processes of TSSs, they can carry proteins, toxins, and factors associated with these systems, reflecting an integrated role in bacterial secretion and virulence, providing future research scopes (Kulp and Kuehn 2010).

**Table 5:** Outer membrane vesicles explored as a vaccine candidate in *Burkhoderia* species

OMVs	Description	References
*B. pseudomallei* strain 1026b OMV vaccine	OMV vaccine safety assessed in rhesus macaques. Upto 100 µg OMV was safe, and immunization produced high titers of OMV, LPS, and CPS-specific plasma IgG	(Petersen et al. 2014)
*B. pseudomallei* strain 1026b OMV vaccine	100 µg OMV immunization induces high titers of OMV-specific serum IgG and IgA; even protecting against lethal aerosol challenges in mice	(Nieves et al. 2011)
*B. pseudomallei* strain Bp82 OMV vaccine *B. mallei* strain China 7 OMV vaccine	Immunization with *B. pseudomallei* OMVs induced the production of *B. mallei*-specific antibodies due to the presence of conserved surface antigens in mice and rhesus macaques	(Baker et al. 2017)
*B. cepacia* ATCC 25416 OMV	A549 cells were treated with 5 µg/mL of OMV along with specific antibiotics that stimulated the expression of pro-inflammatory cytokine IL-1β, IL-6, and TNF-α genes and chemokine IL-8 and MCP-1 genes. In vivo studies using mice was OMV generated at subinhibitory antibiotic concentrations in *B. cepacia* showed correlating observations	(Kim et al. 2020)
*B. pseudomallei OMV* vaccine infection mimicking conditions	OMVs derived from infection-mimicking conditions elicited similar protection to a live-attenuated vaccine, demonstrating strong immunogenicity in mice and suggesting safer alternatives to live vaccines	(Baker et al. 2021)
*B. pseudomallei* OMV vaccine—macaque model	OMV vaccine prevented lung pathology in a rhesus macaque model of pneumonic melioidosis, inducing robust antibody responses with minimal inflammatory risk	(Baker et al. 2025)

### Biofilm formation and intracellular persistence

Biofilm formation is a crucial survival strategy in *B. pseudomallei* and *B. cepacia* complex to confer resistance to antibiotics, the environment and the host immune defenses. Secretion systems converge with quorum sensing and surface-associated structures to regulate biofilm development and persistence (Chin et al. 2015; Borlee et al. 2017). In most of the *Burkholderia* spp., quorum sensing regulates both biofilm formation and virulence. In *B. pseudomallei* and *B. contaminans,* both organisms have shown that secretion systems contribute to colony formation and antimicrobial resistance (Plumley et al. 2017; Kim et al. 2023). Especially in *B. pseudomallei*, T4P and OMVs contribute to biofilm maturation, stability, and dispersal, with specific pili variants upregulated during biofilm development. The bacterium encodes eight different types of T4P, with TFP8 (BPSS2185) being notably upregulated during biofilm maturation and dispersal (Essex-Lopresti et al. 2005). OMVs further enrich the biofilm matrix with outer membrane proteins and lipoproteins, promoting intercellular communication, nutrient acquisition, and host interaction. Genes such as bpss0093 and bps11800 are highly expressed during biofilm formation, suggesting the possibility that they are encoded by OMV-associated proteins (Chin et al. 2015). Together, these secretion-associated structures enhance long-term persistence in both environmental and host-associated niches.

### Antimicrobial resistance and stress adaptation

Intrinsic antimicrobial resistance (AMR) in *Burkholderia* spp. are driven by a combination of structurally modified lipopolysaccharides (LPS), multidrug efflux systems, and secretion-system-mediated stress responses. Modified LPS confers resistance to antimicrobial peptides and polymyxins, where T3SS and T6SS support survival under antibiotic pressure through promoting host cell adaptation and interbacterial competition (Vellasamy et al. 2011). T6SS activity has been linked to the regulation of RND family efflux pumps, inducing expulsion of diverse antibiotics and enhancing resistance in clinical isolates (Scoffone et al. 2015, 2017). Regulatory mutations frequently amplify efflux pump expression, further reinforcing antimicrobial resistance in pathogenic *Burkholderia* strains (Rhodes and Schweizer 2016). In contrast, *B. mallei* exhibits increased antibiotic susceptibility due to genome reduction and loss of resistance determinants (Nierman et al. 2004).

### Host actin dynamics

It is important to note that although T3SS-3 drives endosomal escape in an NFκB-dependent manner, this process occurs independently of any known T3SS-3 effector proteins (Teh et al. 2014). *B. pseudomallei* avoids LC3-mediated autophagy by disrupting actin structures required for autophagosome formation and hence aids in phagosome escape (Allwood et al. 2011). *B. pseudomallei* then employs secretion-system-associated effectors to manipulate host cytoskeleton to facilitate intracellular invasion and motility (Kang et al. 2016). The T3SS effector BipC interacts with host actin to induce actin polymerization leading to enhanced bacterial entry and intracellular movement (Kang et al. 2016). Simultaneously, the Ser/Thr phosphatase family protein BMAA0553 modulates Rho GTPase signaling by interacting with CDC42 and Rho GDP dissociation inhibitor β, thus regulating actin remodeling through altered GTP/GDP cycling (Kang et al. 2016). Alveolar macrophage phagosome escape likely supports cytosolic replication and triggers immune signaling (Lovelace-Macon et al. 2024). These observations indicate that vacuolar escape is the trigger for engaging cytosolic immune sensors, whereas the T3SS-3 machinery itself does not directly activate the NFκB pathway ( Teh et al. 2014). It has been very recently shown that following phagosome escape, flagellin-dependent IL18 release markedly increases more in alveolar macrophages than in monocytes (Lovelace-Macon et al. 2024).

### Functional relevance of secretory systems

Secretion systems involving a single-step translocation, such as T1SS, T3SS, T4SS, and T6SS are known to be associated with virulence as observed in the *B. pseudomallei* complex and *B. cepacia* complex. The T3SS-3 (bsa) cluster mediates direct effector injection into the host cells, facilitating invasion, immune modulation, and intracellular persistence (Suparak et al. 2005). *B. pseudomallei* is known to employ T3SS-secreted cycle-inhibiting factors (Cifs) to activate the ERK pathway, promoting phosphorylation and degradation of pro-apoptotic proteins, leading to suppressed host cell apoptosis (Li et al. 2015). Complementing this, T6SS delivers toxic effectors into both the host cell and the competing bacteria, thereby enhancing adaptability and survival under hostile conditions.

At the effector level, *Burkholderia* spp. T3SS proteins target host ubiquitination and mitochondrial quality-control pathways. BipD induces mitophagy by interacting with the KLHL9/KLHL13/CUL3 E3 ubiquitin ligase complex, promoting ubiquitination of mitofilin and reducing mitochondrial ROS production. Chbp, a cyclomodulin Cif homolog, deamidates ubiquitin and NEDD8, disrupting ubiquitin-mediated signaling, cytoskeletal organization, and cell-cycle progression (Cui et al. 2010; Mariappan et al. 2021). More recently, TssN and TssM have been shown to interact with host ubiquitin pathway components, including polyubiquitin B protein (UBB) and cullin-1a, suggesting cooperative interference with the SCF E3 protein ligase complex. The UBB and cullin-1a subunits were found to interact with TRAF6 and IkBα, which happen to be the targets of TssM. This network of interactions could prove that TssN and TssM together play a role in the deubiquitination of host proteins (Mariappan et al. 2021; Hermanns et al. 2023; Worrall et al. 2023). Collectively, these findings highlight how *Burkholderia* spp. secretion systems operate not merely as transport conduits but as sophisticated virulence platforms that reprogram host cellular processes to promote bacterial persistence and pathogenesis (Nierman et al. 2004; Vellasamy et al. 2011; Scoffone et al. 2015, 2017; Rhodes and Schweizer 2016).

### Clinical implications and translational perspectives

Secretion-system components pose as promising targets for diagnostics and therapeutics in melioidosis and other *Burkholderia-*associated infections. The gold-standard laboratory-diagnostic approach for melioidosis is a conventional culture positivity assay, but it is disadvantageous due to low sensitivity and high turnaround times. Antigenic diversity across *B. pseudomallei* and *B. cepacia* complex strains further complicates vaccine and diagnostic development (Anuntagool et al. 1998; Vesaratchavest et al. 2006; Shafiq et al. 2022). Several serological and molecular assays such as indirect hemagglutination assay (IHA), immunofluorescent assay (IFA), latex agglutination (LA), and PCR-based methods, have been developed but are limited by suboptimal sensitivity, specificity, or infrastructure requirements. The InBiOS active melioidosis detect (AMD) lateral flow assay (LFA) targeting CPS fulfills many ASSURED criteria but exhibits reduced sensitivity in non-blood samples, restricting its universal point-of-care applicability (Sivakumaran et al. 2021). In contrast, secretion-system-derived targets have enabled more specific approaches, such as a nanoparticle-based LFA (MCDA-NB) targeting multiple T3SS-1 regions, demonstrating rapid and sensitive detection of *B. pseudomallei* (Wang et al. 2021a). Beyond T3SS components, antigens of the T6SS-1 protein Hcp1, O-polysaccharide of lipopolysaccharide, GroEL, and conserved glycoproteins arising from the *Burkholderia* O-glycosylation pathway are being explored as diagnostic biomarkers (Wang et al. 2021a). These advances demonstrate that a combination of secretion-system-derived antigens, structural polysaccharides, and conserved proteins would be needed to achieve broad, sensitive, and specific global diagnostic coverage. Moreover, because secretion-system proteins are intimately linked to virulence and host interaction, they hold promise not only as biomarkers for diagnostic approaches but also as vaccine candidates or therapeutic targets to overcome antigenic diversity and improve outcomes in melioidosis and chronic *Burkholderia* spp. infections.

### Concluding remarks and future directions

In conclusion, secretion systems are pivotal for *Burkholderia* spp. pathogenicity, coordinating biofilm persistence, antimicrobial resistance, host manipulation and intracellular survival. While this review encompasses the broader genus, a translational emphasis on *Burkholderia pseudomallei* is essential, as it represents the most clinically significant and well-characterized pathogen within the genus. Thus, integrating molecular insights of the secretion system function with the clinical and ecological perspectives will be critical for identifying conserved virulence determinants and translating these into effective diagnostic and therapeutic strategies. *Burkholderia pseudomallei* possesses a bipartite genome (~4.1 and ~3.2 Mb), a configuration common to pathogenic members including the Bcc and *B. pseudomallei* group. Comparative genomic work across the genus shows that while many pathogenic species (e.g., *B. pseudomallei*, *B. mallei*, *B. thailandensis*) carry two chromosomes, several environmental and plant-associated *Burkholderia* spp. (e.g., *Burkholderia cenocepacia* and *Burkholderia plantarii*) maintain a three-chromosome architecture, reflecting broader metabolic versatility and ecological plasticity (Holden et al. 2004). *Burkholderia* spp. tend to house core housekeeping metabolic genes on chromosome I. The multi-replicon organization, particularly the flexible secondary and tertiary chromosomes, serves as a hotspot for genomic islands, mobile elements, and virulence-associated loci, contributing to niche adaptation, strain diversification, and variability in secretion system gene clusters, especially T3SS and T6SS secretion systems (Holden et al. 2004). This chromosomal organization supports the evolution of complex host-pathogen interaction machinery across *Burkholderia* spp.

Beyond pathogenicity, several *Burkholderia* spp. strains exhibit beneficial traits, such as promoting plant growth, biocontrol activity, environmental adaptability, and the production of antimicrobial secondary metabolites such as antifungals, bacteriocins, and siderophores. These strains also play a critical role in sustainable agriculture, soil health, and bioremediation. These aspects, extensively reviewed by Elshafie and Camele (2021) (Elshafie and Camele 2021), were beyond the current scope of this review article but are important in understanding the genus as a whole.

This review highlights secretion-system-mediated virulence in clinically significant *Burkholderia* spp. pathogens, with particular emphasis on melioidosis and chronic *Burkholderia*-associated infections (White 2003; Meumann et al. 2023). Among secretion systems, T3SS and T6SS emerge as the dominant virulence drivers in pathogenic *Burkholderia* spp. Structural components of secretion systems in *B. pseudomallei* such as conserved translocon proteins of T3SS (e.g., BipD/BipB homologs), ATPase subunits (e.g., BsaS, VirB11), and particularly T3SS-3 contribute to critical effector delivery. These components are less prone to antigenic variation and may therefore provide broader protective or therapeutic coverage (Lara-Tejero and Galán 2019). This maneuveration through secreted and translocated proteins including BopE (GEF-like effector), BopA, BopC, and structural/translocon components such as BsaL/BsaM (Worrall et al. 2023; Costa et al. 2023; Su et al. 2025), positions these proteins as important adjunct targets rather than standalone vaccines due to their variability and context-dependent expression. The T6SS needle/tube protein Hcp1 and effectors such as VgrG showing high conservation, strong immunogenicity, and essential roles in intracellular survival and dissemination, make Hcp1 the most compelling single antigen candidate to date for both diagnostic and therapeutic strategies (Choh et al. 2013; Chieng et al. 2015; Sengyee et al. 2021; Wu et al. 2024). Multiple studies have demonstrated that candidate vaccines against *B. pseudomallei*, including subunit and virulence-associated antigens, fail to induce durable or sterilizing immunity and often confer only partial protection. In parallel, attenuated strains targeting secretion-associated systems, especially T4SS-related mutants have also been explored as an effective vaccine strategy for lethal *Burkholderia* spp. infections (Choh et al. 2013; Johnson and Ainslie 2017; Sengyee et al. 2025). Recent vaccine studies demonstrate stronger and more consistent protection in Hcp1-containing formulations compared to other antigens such as flagellin (FliC), which show limited or partial protective efficacy (Choh et al. 2013; Sengyee et al. 2025). Regulatory proteins such as MprA play central roles in stress response and pathogenic adaptation but are more suitable as adjunct or platform components rather than single antigens.

Given the genus’s extensive antigenic and genomic diversity, integrating conserved secretion system antigens (e.g., Hcp1) into OMV or rationally designed multiepitope platforms represents the most promising path forward for addressing *Burkholderia* spp. infections and advancing globally relevant countermeasures against melioidosis and related infections (Acevedo et al. 2014; Tan et al. 2018). Multicomponent formulations consistently demonstrate improved survival and sterilizing immunity of the host compared to single-antigen approaches (Tapia et al. 2021; Yun et al. 2025). In parallel, small-molecule inhibition of conserved secretion system machinery (e.g., ATPases of T3SS, T2SS, and T4SS) represents an attractive anti-virulence approach that can disarm pathogens without exerting strong selective pressure for resistance (Wang et al. 2021b; Su et al. 2025). Harnessing and advancing these approaches in future may redefine the therapeutic landscape for *Burkholderia*-associated infections.

Future research integrating metabolic and ecological perspectives together with genomic duality, chemical inhibition of secretion systems, and machine-learning-based effector prediction could enhance our understanding of how beneficial and pathogenic traits evolve across the genus, and how this duality could be leveraged to improve medicine and sustainable agricultural practices.

## Data Availability

No datasets were generated or analysed during the current study.
